# Ambient Temperature and the Risk of Preterm Birth in Guangzhou, China (2001–2011)

**DOI:** 10.1289/ehp.1509778

**Published:** 2015-12-15

**Authors:** Jian-Rong He, Yu Liu, Xiao-Yan Xia, Wen-Jun Ma, Hua-Liang Lin, Hai-Dong Kan, Jin-Hua Lu, Qiong Feng, Wei-Jian Mo, Ping Wang, Hui-Min Xia, Xiu Qiu, Louis J. Muglia

**Affiliations:** 1 Division of Birth Cohort Study, and; 2 Department of Health Care, Guangzhou Women and Children’s Medical Center, Guangzhou Medical University, Guangzhou, China; 3 Guangzhou Women and Children’s Health Information Center, Guangzhou, China; 4 Guangdong Provincial Institute of Public Health, Guangdong Provincial Center for Disease Control and Prevention, Guangzhou, China; 5 School of Public Health, Key Lab of Public Health Safety of the Ministry of Education, Fudan University, China; 6 Center for Prevention of Preterm Birth, Cincinnati Children’s Hospital Medical Center, Cincinnati, Ohio, USA

## Abstract

**Background:**

Although effects of weather changes on human health have been widely reported, there is limited information regarding effects on pregnant women in developing countries.

**Objective:**

We investigated the association between maternal exposure to ambient temperature and the risk of preterm birth (< 37 weeks of gestation) in Guangzhou, China.

**Methods:**

We used a Cox proportional hazards model to estimate associations between preterm birth and average temperature during each week of gestation, with weekly temperature modeled as a time-varying exposure during four time windows: 1 week (the last week of the pregnancy), 4 weeks (the last 4 weeks of the pregnancy), late pregnancy (gestational week 20 onward), and the entire pregnancy. Information on singleton vaginal birth between 2001 and 2011 was collected. Daily meteorological data during the same period were obtained from the Guangzhou Meteorological Bureau.

**Results:**

A total of 838,146 singleton vaginal births were included, among which 47,209 (5.6%) were preterm births. High mean temperatures during the 4 weeks, late pregnancy, and the entire pregnancy time windows were associated with an increased risk of preterm birth. Compared with the median temperature (24.4°C), weekly exposures during the last 4 weeks of the pregnancy to extreme cold (7.6°C, the 1st percentile) and extreme heat (31.9°C, the 99th percentile) were associated with 17.9% (95% CI: 10.2, 26.2%) and 10.0% (95% CI: 2.9, 17.6%) increased risks of preterm birth, respectively. The association between extreme heat and preterm birth was stronger for preterm births during weeks 20–31 and 32–34 than those during weeks 35–36.

**Conclusions:**

These findings might have important implications in preventing preterm birth in Guangzhou as well as other areas with similar weather conditions.

**Citation:**

He JR, Liu Y, Xia XY, Ma WJ, Lin HL, Kan HD, Lu JH, Feng Q, Mo WJ, Wang P, Xia HM, Qiu X, Muglia LJ. 2016. Ambient temperature and the risk of preterm birth in Guangzhou, China (2001–2011). Environ Health Perspect 124:1100–1106; http://dx.doi.org/10.1289/ehp.1509778

## Introduction

Preterm birth, defined as birth < 37 completed weeks of gestation, is a complex syndrome and remains a major global public health problem (Goldenberg et al. 2008; Muglia and Katz 2010). It is estimated that approximately 15 million babies were born preterm in 2010 worldwide, accounting for 11% of all live births (Blencowe et al. 2012). Preterm birth is associated with a variety of adverse outcomes, including infant mortality, acute morbidity, long-term neurocognitive disorders, poor growth, respiratory illnesses, behavioral problems, and other complications (Saigal and Doyle 2008). Although a number of risk factors have been explored and relevant interventions have been developed, the incidence of preterm birth has been increasing during the past two decades in most countries (Blencowe et al. 2012), indicating that further research is needed to elucidate its etiology.

Seasonal weather changes have extensive effects on human health outcomes, especially for overall mortality, infectious diseases, and cardiovascular diseases (Woodward et al. 2014). As a specific population, pregnant women might be susceptible to effects of weather changes. Compared with elderly people, however, pregnant women have received less attention. Recently, there is an emerging interest in examining the effects of environmental temperature on the risk of preterm birth (Strand et al. 2011b). Several time-series studies observed that maternal short-term exposure to high temperature was associated with an increased risk of preterm birth (Schifano et al. 2013; Vicedo-Cabrera et al. 2014). On the other hand, negative results were also reported in a few studies (Lee et al. 2008; Wolf and Armstrong 2012). Furthermore, a recent cohort study in Sweden reported that extremely cold temperature was associated with an increased risk of preterm birth (Bruckner et al. 2014). In the context of global climate change and high medical cost for preterm birth, it is necessary to clarify the effects of environmental temperature on the risk of preterm birth.

The most biologically relevant time period for effects of temperature on preterm birth is not known. Previous studies focused mainly on the short-term exposure, which was defined as a few days or weeks before delivery (Basu et al. 2010; Lee et al. 2008; Schifano et al. 2013; Vicedo-Cabrera et al. 2014). Because long-term exposure to temperature might also be relevant for preterm birth (Strand et al. 2011b), we used a Cox proportional hazards model with gestational age as the time axis to evaluate the relationship between short- and long-term temperature exposures and the risk of preterm birth from 2001 to 2011 in Guangzhou, a subtropical city in China. Considering time-dependent exposures, the Cox proportional hazards model has been shown to be useful for evaluating exposure windows for preterm birth (Chang et al. 2012; Suh et al. 2009).

Animal studies have suggested that high temperature exposure could lead to dehydration, reduce uterine blood flow (Stan et al. 2013), and increase the secretion of proinflammatory cytokines and oxytocin (Dreiling et al. 1991; Wolfenson et al. 1993), thereby inducing the uterine contraction and onset of labor. Previous studies also proposed that cold temperature could lead to elevated blood viscosity and vascular constriction (Bruckner et al. 2014) and increase exposure to risk factors of preterm birth, such as passive smoking (Ronchetti et al. 1994) and infectious agents (Murray et al. 2000). These studies suggested that temperature might affect the timing for initiation of spontaneous labor. In the present study, we aimed to examine the effects of temperature on the timing for natural labor. Similar to previous studies (Dadvand et al. 2011; Murray et al. 2000; Schifano et al. 2013; Vicedo-Cabrera et al. 2014), we excluded cesarean section deliveries because they would not reflect physiological mechanisms of temperature on spontaneous birth timing.

## Methods

### Study Population

**Figure 1:**
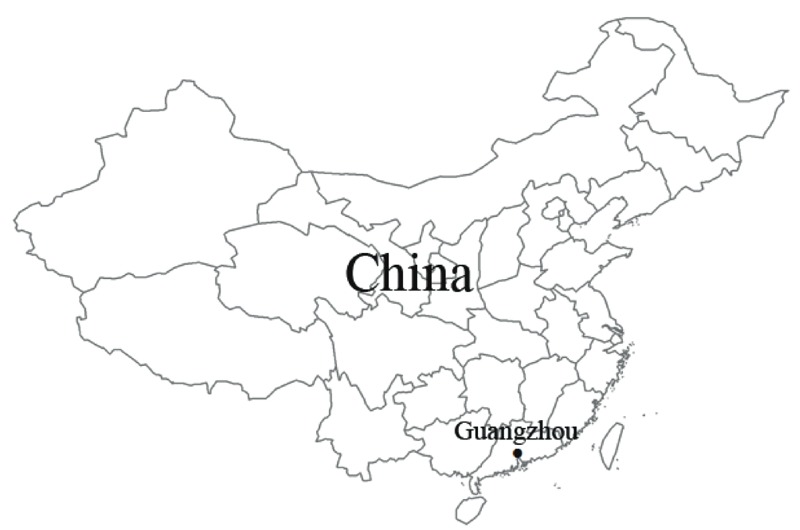
Location of the study area (Guangzhou) in China.

Guangzhou is the largest metropolis in Southern China with a population of 12.9 million (Figure 1). It has a typical subtropical climate with mild winters and hot summers. Data on all singleton births between 1 January 2001 and 31 December 2011 were collected from the Guangzhou Perinatal Health Care and Delivery Surveillance System (GPHCDSS), which was initiated in 2000 and covered > 97% of deliveries in Guangzhou (Guo et al. 2014; He et al. 2014). In the GPHCDSS, information on births is collected from hospitals via computer network and is used to issue birth certificates. The information is confirmed by the chief of midwives and the chief of physicians in hospitals after data entry. When the birth certificate is issued, the Department of Medical Administration and parents also confirm the birth information (Guo et al. 2014; He et al. 2014). Data obtained from GPHCDSS included date of birth, gestational age at birth, birth weight, infant sex, maternal age, educational level, registered residence, and parity. Registered residence represents the permanent residence in the system of household registration (Hukou system) in China, and generally categorized as rural and urban residence. Urban residents find it easier to get access to health services, medical insurance, education, and other social welfare (Wang 2005). The date of conception was calculated by subtracting the gestational age at birth from the date of birth.

All singleton vaginal births at the gestational age of 20–43 weeks were included in the present study. Because the data were collected by the birth date, short pregnancies from the earliest possible conception dates that terminated before the start date of the study (1 January 2001) were not included in the analysis; similarly, long pregnancies from the latest possible conception dates that terminated after the end date of the study (31 December 2011) were also not included in the analysis (Barnett 2011; Strand et al. 2011a; Wolf and Armstrong 2012). Thus, women included at the beginning of the study tended to have longer pregnancies, whereas those included at the end of the study tended to have shorter pregnancies (Barnett 2011; Strand et al. 2011a; Wolf and Armstrong 2012). This phenomenon could lead to artificial seasonal patterns in gestation length and distort the association between time-dependent exposures (e.g., temperature) and outcomes (Barnett 2011; Strand et al. 2011a). To avoid this potential “fixed cohort bias,” we excluded those pregnancies conceived earlier than 20 weeks before the study’s start date and those conceived later than 43 weeks before the study’s end date to ensure that all pregnancies are entirely observable within the study period (Strand et al. 2012; Wolf and Armstrong 2012).

This study was approved by the institutional ethical committee board of Guangzhou Women and Children’s Medical Center. Data used in this study were anonymous, and no individual identifiable information was available for the analysis.

### Outcome Definition

Preterm birth was defined as birth < 37 completed weeks of gestation. Gestational age at birth was determined according to an ultrasound examination in the first or second trimester (Fu and Yu 2011). If the ultrasound examination was unavailable, the last menstrual period (LMP) was used to calculate the gestational age. Preterm birth was also subdivided into three groups: 20–31, 32–34, and 35–36 weeks of gestation.

### Meteorological and Air Pollution Exposure

The daily meteorological data during the study period, including mean temperature (degrees Celsius) and relative humidity (percent), were collected from the Guangzhou Meteorological Bureau. The meteorological data were recorded at a meteorological station in Guangzhou. Daily values for temperature and relative humidity were calculated by averaging four measurements taken at 6-hr intervals over each 24-hr period. Daily air pollution data from 2006–2010 were obtained from Environmental Monitoring Center, including particulate matter with an aerodynamic diameter ≤ 10 μm (PM_10_; micrograms per cubic meter), sulfur dioxide (SO_2_; micrograms per cubic meter), and nitrogen dioxide (NO_2_; micrograms per cubic meter). Air pollution data were measured at a fixed environmental monitoring station located in the center of Guangzhou. The monitoring station providing meteorological and air pollution data follows the quality assurance and quality control programs that were mandated by the Chinese government. These data on temperature and air pollution have been widely used to explore their effects on mortality in Guangzhou (Liu et al. 2014; Xie et al. 2013; Zeng et al. 2014).

We used the estimated date of conception to determine the start and end date of each gestational week for each pregnancy. We then derived the weekly average temperature during each completed gestational week by averaging the daily mean temperature on the last day of the corresponding gestational week and the 6 previous days (Chang et al. 2012). We used the same approach to estimate weekly average values for relative humidity, PM_10_, SO_2_, and NO_2_ for each completed gestational week of pregnancy.

### Statistical Analysis

We used a Cox proportional hazards model with gestational age as the time axis and preterm birth as the outcome. Term births were censored at the end of the 36th week of gestation because they were no longer at risk of being born preterm (Bruckner et al. 2014; Chang et al. 2012). We modeled weekly average temperature as a time-varying independent variable to estimate the hazard of preterm birth during each completed gestational week *t* in association with temperature during four exposure windows: *a*) 1-week (the weekly mean temperature during week *t*); *b*) 4-week (weekly mean temperature for each gestational week from week *t* – 3 through *t;* the 4 weeks are *t* – 3, *t* – 2, *t* – 1, and *t*); *c*) late pregnancy (weekly mean temperature for each gestational week from week 20 through week *t*) and *d*) cumulative (weekly mean temperature for each gestational week from week 1 through week *t*) (Chang et al. 2012; Strand et al. 2012).

We modeled time-varying weekly temperatures during each time window as a cubic spline with 3 degrees of freedom (df) to allow a nonlinear relationship with preterm birth, and plotted the resulting hazard ratios (HRs) and 95% confidence intervals (CIs) to show the relationship over the entire temperature distribution. In addition, we calculated specific HRs relative to the local median temperature (24.4°C) for locally extreme cold (defined as the 1st percentile of temperature, 7.6°C) and moderate cold (the 5th percentile, 11.2°C), and for locally extreme heat (the 99th percentile, 31.9°C) and moderate heat (the 95th percentile, 30.7°C) (Chen et al. 2013). In addition to estimating associations with all preterm births, we performed stratified analyses to estimate associations with three preterm birth subgroups (births during gestational weeks 20–31, 32–34, and 35–36).

Maternal age was categorized into three groups (< 20, 20–34, and > 34 years) and included in the model as a stratified variable (Wang et al. 2013), because we observed that both women aged < 20 and > 34 years had higher risk of preterm birth. The following sociodemographic variables were adjusted for: maternal education level (high school or below vs. college, undergraduate, master or above), parity (primiparity vs. multiparity), and baby’s sex (male vs. female). To control for long-term trend and seasonality, we included the year of conception and the month of conception as indicator variables. Similar to the temperature, the relative humidity was included as a time-dependent variable at each exposure window using a spline with 3 df.

Sensitivity analyses were performed to evaluate the robustness of results. We included the month of conception as a spline with 3 to 6 df. For pregnancies that took place during 2006–2010, we performed separate models adjusted for weekly average concentrations of PM_10_, SO_2_, and NO_2_ as time-varying covariates, using time windows corresponding to those used for temperature. However, because air pollution exposures might be causal intermediates between temperature and preterm birth, adjustment may block part of the total effects of temperature (Buckley et al. 2014). Finally, we used a quasi-Poisson regression with distributed lag nonlinear model (DLNM) as an alternative approach to estimate the cumulative effects of low and high temperatures on preterm birth for single-day lags (lag 0, representing temperature on the same day of preterm birth, up to 27-day lag) and during three cumulative lag periods (lag 0–6, 0–13, and 0–27 days, where lag 0–6 represents the temperature on the day of preterm birth and the previous 6 days, and so forth) (Gasparrini et al. 2010). The model used for analysis was

Log[*E*(*Y_t_*)] = α + β*Temp_t_*_,_ *_l_* + *NS*(*RH_t_*, *5*) + *NS*(*Time, 5/year*) + γ*DOW_t_* + δLog(*Z_t_*),

where *t* represents the day of the observation; *Y_t_* is the observed number of preterm births on day *t*; *Temp_t,l_* is a matrix representing the two-dimensional relationship of mean temperature and lag days; *l* denotes the maximum lag days; *NS*(*RH_t_, 5*) is a natural cubic spline with 5 df for relative humidity; *NS*(*Time, 5/year*) is a natural cubic spline with 5 df/year for long-term trend and seasonality; and *DOW_t_* is the day of the week (a categorical variable with Sunday as the reference). *Z_t_* is a daily expected count of preterm births on day *t*:


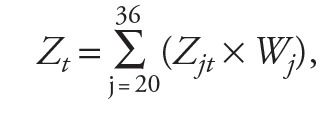


where *Z_jt_* is the number of pregnancies at risk during gestational week *j* (ranging from 20 through 36 weeks) on day *t*, and *W_j_* is the conditional probability of birth during gestational week *j*, as proposed by Vicedo-Cabrera et al. (2014). The quasi-Akaike information criterion (QAIC) was used to choose the df for temperature and lags (Gasparrini et al. 2010; Peng et al. 2006), and the final model used a natural cubic spline with 4 df to estimate the nonlinear effect of temperature, and 4 df to estimate the lag effects. We plotted relative risks (RRs) across the entire temperature distribution for single-day lags, and estimated specific RRs for extremely low and high temperatures relative to the median (24.4°C) for the three cumulative lags.

All statistical analyses were performed using SAS statistical software version 9.3 (SAS Institute Inc., Cary, NC) and the R statistical environment (version 2.15) (R Core Team 2013) with the smoothHR (Meira-Machado et al. 2013) and DLNM packages.

## Results

A total of 1,420,348 singleton births at the gestational age of 20–43 weeks were recorded during the study period. After exclusion of 582,202 cesarean section births (with preterm birth rate of 5.2%), 838,146 singleton vaginal births were included in this study. Among them, 47,209 (5.6%) were preterm births, with a daily average of 11.8 preterm births. The demographic characteristics of the study population are shown in Table 1. Compared with the mothers of term babies, those of preterm babies tended to be primiparous and have higher percentage of age < 20 and > 34 years. Male infants were more likely to be born preterm. Preterm babies had a slightly higher percentage of being born in winter (December–February) (Table 1). The distributions of educational level and registered residence were similar between the preterm birth and term birth groups (Table 1).

**Table 1:** Characteristics of study population (2001–2011) [*n* (%)].

Characteristics	Preterm birth	Term birth
Total	47,209 (5.6)	790,937 (94.4)
Maternal age (years)
< 20	2,415 (5.2)	27,707 (3.5)
20–34	41,017 (88.8)	719,379 (91.9)
> 34	2,738 (5.9)	35,613 (4.6)
Missing	1,039	8,238
Mean ± SD	26.3 ± 4.7	26.3 ± 4.4
Educational level
High school or below	41,040 (86.9)	684,596 (86.6)
College	3,865 (8.2)	66,272 (8.4)
Undergraduate	2,037 (4.3)	35,079 (4.4)
Master or above	267 (0.6)	4,990 (0.6)
Registered residence^*a*^
Rural	12,411 (26.5)	207,030 (26.3)
Urban	34,430 (73.5)	580,283 (73.7)
Missing	368	3,624
Parity
Primiparity	32,404 (68.6)	510,836 (64.6)
Multiparity	14,805 (31.4)	280,101 (35.4)
Baby’s sex
Male	27,686 (58.7)	418,262 (52.9)
Female	19,523 (41.4)	372,675 (47.1)
Birth weight (g)
Mean ± SD	2,419 ± 529	3,193 ± 381
Season of birth
December–February	11,836 (25.1)	188,216 (23.8)
March–May	9,677 (20.5)	165,003 (20.9)
June–August	12,198 (25.8)	202,342 (25.6)
September–November	13,498 (28.6)	235,376 (29.8)
Preterm birth subgroups (weeks of gestation)
20–31	4,988 (10.6)
32–34	11,323 (24.0)
35–36	30,898 (65.5)
^***a***^Registered residence represents the permanent residence in the system of household registration (Hukou system) in China.		

**Figure 2:**
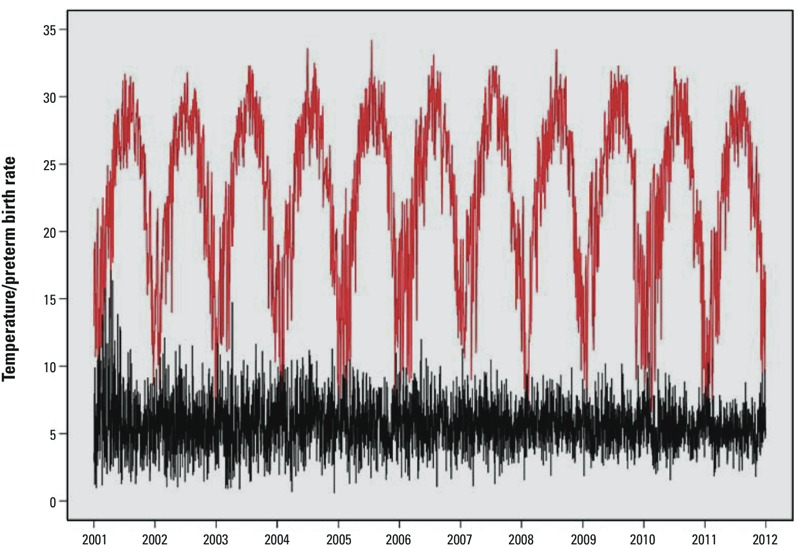
Daily temperature (°C) and preterm birth rate (%) over the study period. The red line represents the temperature, and the black line represents the preterm birth rate.

The median daily mean temperature and relative humidity were 24.4°C and 74% during the study period. The Spearman’s correlations showed a weak correlation between mean temperature and relative humidity (*r_s_* = 0.098). Figure 2 shows the 11-year time series of temperature and preterm birth rate, which was calculated by dividing daily counts of preterm birth by daily births. The daily mean temperature was lowest in December and January and peaked in July and August (Figure 2). The daily rate of preterm births appeared to be relatively high in January to March, although the yearly variations were not completely consistent over the study period (Figure 2).

**Figure 3:**
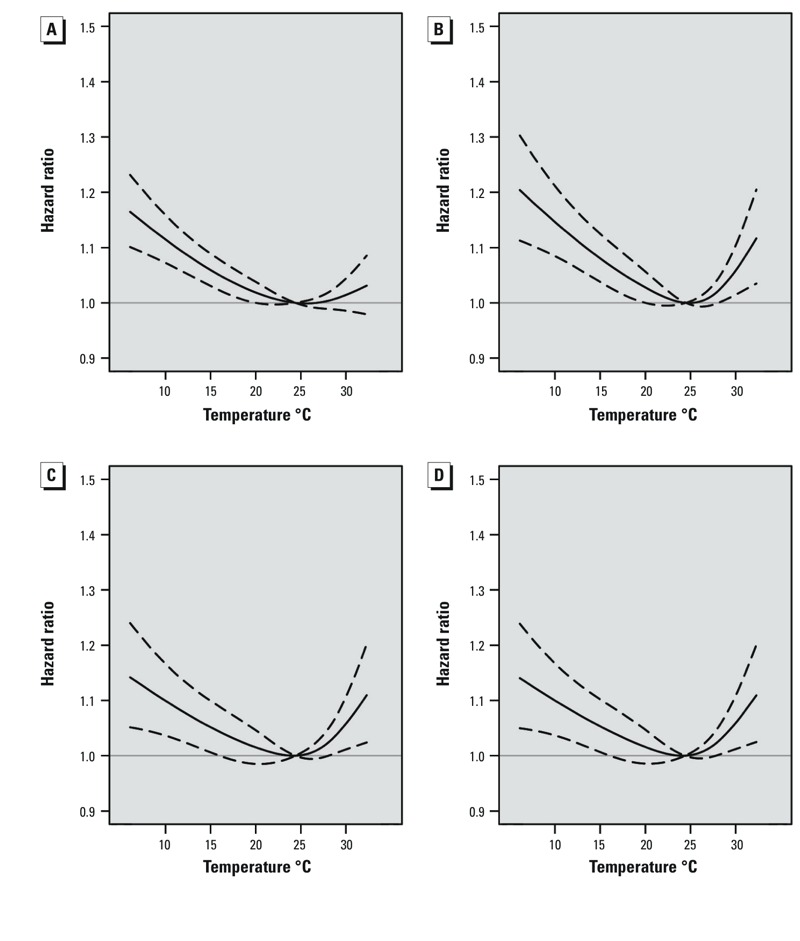
Adjusted hazard ratios (solid line) and 95% confidence intervals (dashed lines) for preterm birth in association with weekly average temperature modeled as a time-dependent variable during four time windows: 1-week (*A*), 4-week (*B*), late pregnancy (*C*), and cumulative (*D*). Estimates are relative to the median temperature for the study area (24.4°C). All values are based on Cox proportional hazards models with gestational age as the underlying time axis and adjusted for maternal age, education, parity, baby’s sex, year and month of conception, and relative humidity (during the corresponding time window).

Figure 3 shows adjusted HRs for weekly mean temperatures, relative to 24.4°C, during different exposure windows. Associations were U-shaped, with significant associations between preterm birth risk and cold temperatures during all four time windows, and significant associations with high temperatures during the 4-week, late pregnancy (≥ 20 weeks), and cumulative (entire pregnancy) time windows, but not the 1-week time window. Associations with extremely and moderately low and high temperatures were strongest for temperature during the 4-week time window, and HRs for low temperatures were larger than corresponding HRs for high temperatures during all time windows (Table 2). For example, relative to 24.4°C, exposures to extreme cold (7.6°C) and extreme heat (31.9°C) during the 4-week time window were associated with 17.9% (95% CI: 10.2, 26.2%) and 10.0% (95% CI: 2.9, 17.6%) higher risks of preterm birth, respectively.

**Table 2:** Adjusted hazard ratios (95% CI) for preterm birth in association with low and high temperatures during different time windows of pregnancy.

Temperature^*a*^	Hazard ratios (95% CIs)^*b*^			
	1-week	4-week	Late pregnancy	Cumulative
Extreme cold	1.143 (1.090,1.200)	1.179 (1.102,1.262)	1.124 (1.046,1.207)	1.123 (1.046,1.207)
Moderate cold	1.100 (1.063,1.140)	1.129 (1.074,1.187)	1.087 (1.030,1.148)	1.088 (1.031,1.148)
Moderate heat	1.019 (0.984,1.056)	1.075 (1.020,1.132)	1.073 (1.016,1.133)	1.074 (1.016,1.135)
Extreme heat	1.026 (0.981,1.074)	1.100 (1.029,1.176)	1.095 (1.021,1.175)	1.096 (1.022,1.175)
^***a***^Extreme cold, moderate cold, moderate heat, and extreme heat were defined as local 1st (7.6°C), 5th (11.2°C), 95th (30.7°C), and 99th (31.9°C) percentile temperature over an 11-year period, respectively. ^***b***^Hazard ratios are based on Cox proportional hazards models with average weekly temperatures modeled as time-dependent variables for exposures during four time windows: 1-week (average weekly temperature during the current week), 4-week (temperature during the current and 3 previous weeks), late pregnancy (average weekly temperatures for gestational week 20 through week 36 or birth), and cumulative (average weekly temperatures for gestational week 1 through week 36 or birth). Data for term births are censored after the 36th week of pregnancy. Estimates are relative to the median temperature for the study area (24.4°C). The following variables were adjusted for: maternal age, education, and parity, baby’s sex, year and month of conception, and relative humidity (during the corresponding time window).				

**Figure 4:**
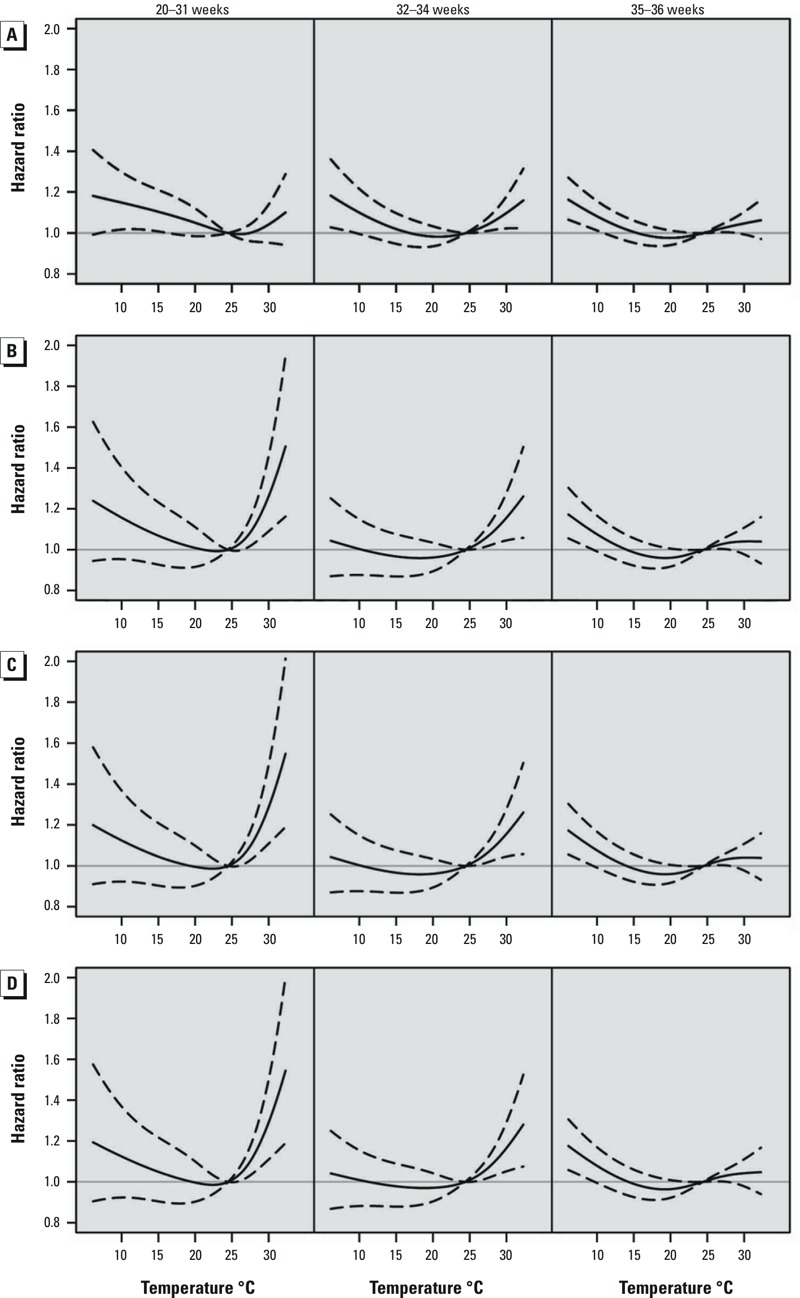
Adjusted hazard ratios (solid line) and 95% confidence intervals (dashed lines) for preterm birth (during weeks 20–31, 32–34, and 35–36) in association with weekly average temperature modeled as a time-dependent variable during four time windows: 1-week (*A*), 4-week (*B*), late pregnancy (*C*), and cumulative (*D*). Estimates are relative to the median temperature for the study area (24.4°C). All values are based on Cox proportional hazards models with gestational age as the underlying time axis and adjusted for maternal age, education, parity, baby’s sex, year and month of conception, and relative humidity (during the corresponding time window).

Figure 4 presents the stratified relationship of preterm birth subgroups with the mean temperature. Generally, the shapes of stratified relationships were similar to those in the overall analysis. Associations with higher temperatures were stronger for preterm births during weeks 20–31 (*n* = 4,988 preterm births) and 32–34 (*n* = 11,323) compared with preterm births during weeks 35–36 (*n* = 30,898), though estimates for later preterm births were more precise, at least in part due to larger numbers of observations (Figure 4). Specifically, the HRs (95% CIs) of extremely high temperature for preterm births during weeks 20–31 and 32–34 were 1.45 (95% CI: 1.15, 1.83) and 1.29 (95% CI: 1.08, 1.54) at the 4-week time window, respectively, and the HR during weeks 35–36 was 1.08 (95% CI: 0.97, 1.21).

In the sensitivity analyses, we included the month as a spline with 3–6 df, and the effect estimates for temperature remained similar (see Table S1). HRs for temperature and preterm among pregnancies during 2006–2010 (*n* = 450,230, including 25,329 preterm births) after adjustment for weekly average PM_10_, SO_2_, and NO_2_ concentrations during time windows corresponding to those for temperature were similar to those for overall study population, although HRs were slightly attenuated and less precise after adjustment. However, these differences may be attributable at least partly to the use of a different population (2006–2010 vs. 2001–2011), rather than to effects of adjustment for air pollutants per se (see Figure S1).

Associations between preterm and temperature also tended to be U-shaped when estimated using a quasi-Poisson regression with DLNM (see Figure S2). Associations with extreme cold and extreme heat both were strongest for temperature on the same day of preterm birth (lag 0) and null after 4- and 6-day lags, respectively. The association with extreme cold was strongest for the longest cumulative lag (lag 0–27), whereas HRs for extreme heat were > 1 at lag 0–6 but < 1 for longer cumulative lags (see Table S2).

## Discussion

In the present study, both low and high temperatures during pregnancy were associated with an increased risk of preterm birth. Extreme cold temperatures were associated with the risk of preterm birth during all time windows examined (1-week, 4-week, late pregnancy, and entire pregnancy), and extreme heat was associated with preterm birth risk during all but the shortest time window of exposure. Furthermore, extreme heat was more strongly associated with preterm births during 20–31 and 32–34 weeks of gestation than with preterm births during weeks 35–36.

To our knowledge, this study may be the first one to investigate the effects of ambient temperature on the risk of preterm birth in developing countries (Beltran et al. 2013). Air conditioning or central heating is less used in developing countries than in developed countries; thus the population may be more susceptible to climate change, and the harmful effects of extreme temperature on health may be more pronounced. Using the time-to-event model, we also extended earlier work by examining different temperature exposure windows, including both short- and long-term exposures. Moreover, our study has a larger sample size compared with previous studies, which enabled us to evaluate expected subtle effects of temperature.

The prevalence of preterm birth among singleton in the study area (5.6% for vaginal delivery, 5.2% for cesarean section delivery) was similar to those of previous reports in Guangzhou (5.1% to ~ 6.4% from 2002 to 2012) (Fu and Yu 2011; Guo et al. 2014), Korea (5.3% in 2008) (Park et al. 2013), and Japan (5.7% in 2012) (Ministry of Health, Labour and Welfare, Japan 2012), which were lower than that of the United States (9.7% in 2013) (Martin et al. 2015). The rate of preterm birth in China is lower than that of some Western countries. This may be explained by several factors, including genetic predisposition (black women tend to have higher rates of preterm birth than other ethnic groups) (Goldenberg et al. 2008), early prenatal identification and termination of pregnancies with significant fetal anomalies that might otherwise result in a preterm birth (Purisch et al. 2008), and lower prevalence of risk factors, such as maternal advanced age and active smoking (Beck et al. 2010; Goldenberg et al. 2008). Furthermore, we did not observe significant differences in maternal educational level and status of registered residence between preterm birth and term birth subjects. This might be explained partly by implementation of several public health programs to promote maternal and children’s health in Guangzhou, which improved the equality for access to antenatal care service among different social economic statuses.

Few studies have explored the effects of ambient temperature on the rate of preterm birth with inconsistent results (Strand et al. 2011b). Two large studies, conducted in London, United Kingdom, and in two German states did not find any association between temperature and the risk of preterm birth (Lee et al. 2008; Wolf and Armstrong 2012). Nevertheless, a study in Rome, Italy, reported that a 1°C increase in maximum apparent temperature at lag 0–2 resulted in an increase of 1.9% in daily preterm birth in warm season (Schifano et al. 2013). In addition, a study in California showed that every 5.6°C increase in apparent temperature at lag 0–6 increased preterm births by 8.6% (Basu et al. 2010). In the present study, we observed a U-shaped relationship between temperature and preterm birth over the whole year. This finding was in accordance with a recent study in Sweden, which also found that both cold and warm temperature extremes were associated with an increased risk of preterm birth (Bruckner et al. 2014). Furthermore, we observed that an association of extreme cold with preterm birth risk was stronger than that with extreme heat, although the low temperature is mild in our study region. This result might be explained by the fact that the population in Guangzhou, a subtropical city, is less accustomed to cold weather, and thus more susceptible to cold stress. In addition, central heating is rarely used in subtropical areas such as Guangzhou, which may lead to greater adverse effects of extremely cold weather on health (Xie et al. 2013).

There were several potential reasons for differences between our findings and those of previous studies. First, the range of climatic conditions, socioeconomic level, and population susceptibility may vary across areas. In addition, we speculate that the study design, statistical methods, exposure windows, and sample size are important factors (Strand et al. 2011b). For example, previous studies have examined nonlinear relationships using logistic regression (Basu et al. 2010; Lee et al. 2008; Wolf and Armstrong 2012), Cox proportional hazards model (Bruckner et al. 2014; Strand et al. 2012), and Poisson generalized additive model (Schifano et al. 2013), whereas the delayed effects have been estimated using single-day lags and moving average lags (Basu et al. 2010), distributed lag model (Schifano et al. 2013), or distributed lag nonlinear models (Vicedo-Cabrera et al. 2014). In the present study, estimates from both the Cox proportional hazards model and the quasi-Poisson regression with DLNM indicated U-shaped associations between preterm and mean temperature, though associations based on the quasi-Poisson regression with DLNM were strongest for recent exposures (single-day lags < 1 week), whereas high temperatures during the 1-week time window were not significantly associated with preterm based on the Cox proportional hazards model. This indicated that different statistical methods used might result in different estimates, even in the same study population. Compared with other statistical methods, the Cox proportional hazards model has several advantages. For example, considering time-varying exposures, the time-to-event analysis could effectively evaluate both long- and short-term exposure windows (Chang et al. 2012; Suh et al. 2009). Furthermore, the time-dependent survival analysis could also eliminate potential confounding, which is caused by seasonal variation in the gestational age distribution of pregnancies at risk (< 37 weeks) and the increasing probability of birth with higher gestational age (Darrow et al. 2009), by comparing fetuses at the same gestational age (Strand et al. 2012). Therefore, the Cox proportional hazards model has been recommended for future studies of time-dependent exposures on preterm birth (Chang et al. 2012; Strand et al. 2012).

Biological mechanisms that might contribute to the association between ambient temperature and preterm birth are unclear. Previous studies proposed several hypotheses. High temperature exposure causes dehydration and reduces uterine blood flow in pregnant women, which can induce the uterine contraction and onset of labor (Basu et al. 2010; Stan et al. 2013). In addition, animal studies showed that heat exposure may increase the secretion of proinflammatory cytokines and oxytocin to induce labor (Dadvand et al. 2011; Dreiling et al. 1991; Wolfenson et al. 1993). Furthermore, high temperature may increase blood viscosity and cholesterol levels, which may be risk factors of preterm birth (Basu et al. 2010). In our study population, extreme cold (7.6°C) also was associated with preterm birth. There are several explanations for the association with low temperature. Previous studies showed that cold temperature could increase exposure to passive smoking (Ronchetti et al. 1994) and infectious agents (Murray et al. 2000), and result in occurrence of preeclampsia and pregnancy-induced hypertension (Bider et al. 1991), all of which are potential risk factors for preterm birth (Goldenberg et al. 2008; Muglia and Katz 2010). In addition, cold temperature could also lead to increased blood viscosity and vascular constriction (Bruckner et al. 2014), and thus might be associated with an increased risk of preterm birth (Basu et al. 2010). Taken together, there are many potential pathways through which temperature can affect the risk of preterm birth. Further studies are needed to elucidate the underlying mechanisms.

Several limitations should be mentioned. First, we used data from one meteorological station to define the exposure for all women in Guangzhou. This could cause exposure misclassification. Second, we were unable to distinguish spontaneous preterm birth from iatrogenic preterm birth, although we restricted the study population to those with vaginal delivery. Exposure to temperature might have different effects on these subtypes of preterm birth. We have no data on the percentages of identification method of gestational age (LMP, clinical estimate, ultrasound) and their changes during the study period, which might influence the incidence of early preterm birth and late preterm birth. In Guangzhou, it is a routine practice to confirm gestational age using ultrasound examination in the first or second trimester (Fu and Yu 2011). However, some clinicians in poor rural areas might still be using the LMP-based or clinical estimation–based methods (estimated as < 10% of all births). We also did not have information on the history of preterm delivery and pregnancy complications, all of which are potential risk factors for preterm birth. Finally, we are unable to distinguish the consequences of the temperature itself with other factors associated with changes in weather, such as nutrition, activity, and infectious exposures.

## Conclusions

Exposure to both low and high temperatures during pregnancy was associated with an increased risk of preterm birth in our study population. These findings might have important implications in preventing preterm birth in Guangzhou as well as other areas with similar variations in weather conditions.

## Supplemental Material

(1.7 MB) PDF

## References

[r1] Barnett AG 2011 Time-dependent exposures and the fixed-cohort bias [Letter]. Environ Health Perspect 119 A422 A423, doi:10.1289/ehp.1103885 21968256 PMC3230453

[r2] Basu R, Malig B, Ostro B (2010). High ambient temperature and the risk of preterm delivery.. Am J Epidemiol.

[r3] Beck S, Wojdyla D, Say L, Betran AP, Merialdi M, Requejo JH (2010). The worldwide incidence of preterm birth: a systematic review of maternal mortality and morbidity.. Bull World Health Organ.

[r4] Beltran AJ, Wu J, Laurent O (2013). Associations of meteorology with adverse pregnancy outcomes: a systematic review of preeclampsia, preterm birth and birth weight.. Int J Environ Res Public Health.

[r5] Bider D, Sivan E, Seidman DS, Dulitzky M, Mashiach S, Serr DM (1991). Meteorological factors in hypertensive disorders, vaginal bleeding and premature rupture of membranes during pregnancy.. Gynecol Obstet Invest.

[r6] Blencowe H, Cousens S, Oestergaard MZ, Chou D, Moller AB, Narwal R (2012). National, regional, and worldwide estimates of preterm birth rates in the year 2010 with time trends since 1990 for selected countries: a systematic analysis and implications.. Lancet.

[r7] Bruckner TA, Modin B, Vågerö D (2014). Cold ambient temperature *in utero* and birth outcomes in Uppsala, Sweden, 1915–1929.. Ann Epidemiol.

[r8] Buckley JP, Samet JM, Richardson DB (2014). Commentary: does air pollution confound studies of temperature?. Epidemiology.

[r9] Chang HH, Reich BJ, Miranda ML (2012). Time-to-event analysis of fine particle air pollution and preterm birth: results from North Carolina, 2001–2005.. Am J Epidemiol.

[r10] Chen R, Wang C, Meng X, Chen H, Thach TQ, Wong CM (2013). Both low and high temperature may increase the risk of stroke mortality.. Neurology.

[r11] Dadvand P Basagaña X Sartini C Figueras F Vrijheid M de Nazelle A 2011 Climate extremes and the length of gestation. Environ Health Perspect 119 1449 1453, doi:10.1289/ehp.1003241 21659038 PMC3230440

[r12] Darrow LA, Strickland MJ, Klein M, Waller LA, Flanders WD, Correa A (2009). Seasonality of birth and implications for temporal studies of preterm birth.. Epidemiology.

[r13] Dreiling CE, Carman FS, Brown DE (1991). Maternal endocrine and fetal metabolic responses to heat stress.. J Dairy Sci.

[r14] Fu J, Yu M (2011). A hospital-based birth weight analysis using computerized perinatal data base for a Chinese population.. J Matern Fetal Neonatal Med.

[r15] Gasparrini A, Armstrong B, Kenward MG (2010). Distributed lag non-linear models.. Stat Med.

[r16] Goldenberg RL, Culhane JF, Iams JD, Romero R (2008). Epidemiology and causes of preterm birth.. Lancet.

[r17] Guo Y Liu Y He JR Xia XY Mo WJ Wang P 2014 Changes in birth weight between 2002 and 2012 in Guangzhou, China. PLoS One 9 e115703, doi:10.1371/journal.pone.0115703 25531295 PMC4274089

[r18] He JR, Xia HM, Liu Y, Xia XY, Mo WJ, Wang P (2014). A new birthweight reference in Guangzhou, southern China, and its comparison with the global reference.. Arch Dis Child.

[r19] Lee SJ, Hajat S, Steer PJ, Filippi V (2008). A time-series analysis of any short-term effects of meteorological and air pollution factors on preterm births in London, UK.. Environ Res.

[r20] Liu T, Zhang YH, Xu YJ, Lin HL, Xu XJ, Luo Y (2014). The effects of dust-haze on mortality are modified by seasons and individual characteristics in Guangzhou, China.. Environ Pollut.

[r21] Martin JA, Hamilton BE, Osterman MJ, Curtin SC, Matthews TJ (2015). Births: final data for 2013.. Natl Vital Stat Rep.

[r22] Meira-Machado L Cadarso-Suárez C Gude F Araújo A 2013 smoothHR: an R package for pointwise nonparametric estimation of hazard ratio curves of continuous predictors. Comput Math Methods Med 2013 745742, doi:10.1155/2013/745742 24454541 PMC3876718

[r23] Ministry of Health, Labour and Welfare, Japan (2012). Statistics & Other Data. Vital Statistics. Statistics and Information Department.. http://www.e-stat.go.jp/SG1/estat/ListE.do?lid=000001112798.

[r24] Muglia LJ, Katz M (2010). The enigma of spontaneous preterm birth.. N Engl J Med.

[r25] Murray LJ, O’Reilly DP, Betts N, Patterson CC, Davey Smith G, Evans AE (2000). Season and outdoor ambient temperature: effects on birth weight.. Obstet Gynecol.

[r26] Park MJ, Son M, Kim YJ, Paek D (2013). Social inequality in birth outcomes in Korea, 1995–2008.. J Korean Med Sci.

[r27] Peng RD, Dominici F, Louis TA (2006). Model choice in time series studies of air pollution and mortality.. J R Stat Soc Ser A Stat Soc.

[r28] Purisch SE, DeFranco EA, Muglia LJ, Odibo AO, Stamilio DM (2008). Preterm birth in pregnancies complicated by major congenital malformations: a population-based study.. Am J Obstet Gynecol.

[r29] R Core Team (2013). R: A Language and Environment for Statistical Computing.. http://www.R-project.org/.

[r30] Ronchetti R, Bonci E, de Castro G, Signoretti F, Macrì F, Ciofetta GC (1994). Relationship between cotinine levels, household and personal smoking habit and season in 9–14 year old children.. Eur Respir J.

[r31] Saigal S, Doyle LW (2008). An overview of mortality and sequelae of preterm birth from infancy to adulthood.. Lancet.

[r32] Schifano P, Lallo A, Asta F, De Sario M, Davoli M, Michelozzi P (2013). Effect of ambient temperature and air pollutants on the risk of preterm birth, Rome 2001–2010.. Environ Int.

[r33] Stan CM Boulvain M Pfister R Hirsbrunner-Almagbaly P 2013 Hydration for treatment of preterm labour. Cochrane Database Syst Rev 11 CD003096, doi:10.1002/14651858.CD003096.pub2 24190310 PMC11751767

[r34] Strand LB Barnett AG Tong S 2011a Methodological challenges when estimating the effects of season and seasonal exposures on birth outcomes. BMC Med Res Methodol 11 49, doi:10.1186/1471-2288-11-49 21501523 PMC3102035

[r35] Strand LB, Barnett AG, Tong S (2011b). The influence of season and ambient temperature on birth outcomes: a review of the epidemiological literature.. Environ Res.

[r36] Strand LB, Barnett AG, Tong S (2012). Maternal exposure to ambient temperature and the risks of preterm birth and stillbirth in Brisbane, Australia.. Am J Epidemiol.

[r37] Suh YJ, Kim H, Seo JH, Park H, Kim YJ, Hong YC (2009). Different effects of PM_10_ exposure on preterm birth by gestational period estimated from time-dependent survival analyses.. Int Arch Occup Environ Health.

[r38] Vicedo-Cabrera AM, Iñíguez C, Barona C, Ballester F (2014). Exposure to elevated temperatures and risk of preterm birth in Valencia, Spain.. Environ Res.

[r39] Wang FL (2005). Organizing through Division and Exclusion: China’s Hukou System..

[r40] Wang J, Williams G, Guo Y, Pan X, Tong S (2013). Maternal exposure to heatwave and preterm birth in Brisbane, Australia.. BJOG.

[r41] Wolf J Armstrong B 2012 The association of season and temperature with adverse pregnancy outcome in two German states, a time-series analysis. PLoS One 7 e40228, doi:10.1371/journal.pone.0040228 22792247 PMC3391296

[r42] Wolfenson D, Bartol FF, Badinga L, Barros CM, Marple DN, Cummins K (1993). Secretion of PGF2α and oxytocin during hyperthermia in cyclic and pregnant heifers.. Theriogenology.

[r43] Woodward A, Smith KR, Campbell-Lendrum D, Chadee DD, Honda Y, Liu Q (2014). Climate change and health: on the latest IPCC report.. Lancet.

[r44] Xie H Yao Z Zhang Y Xu Y Xu X Liu T 2013 Short-term effects of the 2008 cold spell on mortality in three subtropical cities in Guangdong Province, China. Environ Health Perspect 121 210 216, doi:10.1289/ehp.1104541 23128031 PMC3569675

[r45] Zeng W, Lao X, Rutherford S, Xu Y, Xu X, Lin H (2014). The effect of heat waves on mortality and effect modifiers in four communities of Guangdong Province, China.. Sci Total Environ.

