# Supplementary material for: Ambient Temperature and the Risk of Preterm Birth in Guangzhou, China (2001–2011)
Source: Environ Health Perspect. 2015 Dec 15;124(7):1100–6. doi: 10.1289/ehp.1509778 (PMC4937853; doi:10.1289/ehp.1509778)
Supplement: (1.7 MB) PDF [file ehp.1509778.s001.acco.pdf]

Environ Health Perspect
DOI: 10.1289/ehp.1509778

Note to readers with disabilities: *EHP* strives to ensure that all journal content is accessible to all readers. However, some figures and Supplemental Material published in *EHP* articles may not conform to 508 standards due to the complexity of the information being presented. If you need assistance accessing journal content, please contact ehp508@niehs.nih.gov. Our staff will work with you to assess and meet your accessibility needs within 3 working days.

Supplemental Material

Ambient Temperature and the Risk of Preterm Birth in Guangzhou, China (2001–2011)

Jian-Rong He, Yu Liu, Xiao-Yan Xia, Wen-Jun Ma, Hua-Liang Lin, Hai-Dong Kan, Jin-Hua Lu, Qiong Feng, Wei-Jian Mo, Ping Wang, Hui-Min Xia, Xiu Qiu, and Louis J. Muglia

Table of Contents

Table S1. Hazard ratios (95% confidence intervals) of low and high temperature compared to the median temperature (24.4 °C) when the month of conception was included as a spline in the model.

Table S2. Cumulative effects of low and high temperatures using quasi-Poisson regression with distributed lag non-linear model.

Figure S1. Adjusted hazard ratios (solid line) and 95% confidence intervals (dashed lines) for preterm birth in association with weekly average temperature modeled as a time-dependent variable during 4 time windows after adjustment for PM10, SO2 and NO2 during 2006-2010: 1-week (A), 4-week (B), late pregnancy (C), and cumulative (D). Estimates are relative to the median temperature for the study area (24.4 °C). All values are based on Cox proportional hazards models with gestational age as the underlying time axis and adjusted for maternal age, education, parity, baby's sex, year and month of conception, relative humidity and air pollutants (during the corresponding time window).

Figure S2. Relationship between mean temperature and preterm birth using quasi-Poisson regression with distributed lag non-linear model. Estimates are relative to the median temperature for the study area (24.4 °C). The following variables were adjusted for: relative

humidity (during the corresponding day), long-term trend and seasonality, day of the week, and daily expected count of preterm births on the corresponding day. Lags represents single-day lags (lag 0, representing temperature on the same day of preterm birth, up to 27-day lag).

Table S1. Hazard ratios (95% confidence intervals) of low and high temperature compared to the median temperature (24.4 ^{o}C) when the month of conception was included as a spline in the model.

Exposure periods[a]	Temperature[b]	Degrees of freedom for splines of month			
		3	4	5	6
1-week	Extreme cold	1.127 (1.082, 1.174)	1.154 (1.105, 1.205)	1.147 (1.094, 1.204)	1.140 (1.088, 1.195)
	Moderate cold	1.086 (1.055, 1.118)	1.109 (1.074, 1.145)	1.104 (1.066, 1.144)	1.102 (1.064, 1.141)
	Moderate heat	1.016 (0.982, 1.051)	1.013 (0.980, 1.048)	1.011 (0.976, 1.046)	1.019 (0.984, 1.056)
	Extreme heat	1.021 (0.978, 1.066)	1.020 (0.977, 1.065)	1.016 (0.972, 1.062)	1.028 (0.982, 1.075)
4-week	Extreme cold	1.158 (1.093, 1.227)	1.206 (1.135, 1.281)	1.188 (1.110, 1.271)	1.176 (1.100, 1.257)
	Moderate cold	1.105 (1.060, 1.152)	1.146 (1.096, 1.199)	1.133 (1.078, 1.191)	1.131 (1.076, 1.189)
	Moderate heat	1.068 (1.017, 1.121)	1.063 (1.013, 1.116)	1.057 (1.005, 1.112)	1.074 (1.020, 1.131)
	Extreme heat	1.088 (1.022, 1.157)	1.086 (1.020, 1.155)	1.076 (1.009, 1.149)	1.101 (1.030, 1.177)
Late pregnancy	Extreme cold	1.120 (1.052, 1.193)	1.174 (1.101, 1.252)	1.134 (1.056, 1.218)	1.123 (1.046, 1.206)
	Moderate cold	1.074 (1.024, 1.126)	1.120 (1.066, 1.176)	1.092 (1.034, 1.152)	1.090 (1.032, 1.151)
	Moderate heat	1.071 (1.018, 1.127)	1.067 (1.014, 1.123)	1.051 (0.996, 1.109)	1.072 (1.015, 1.132)
	Extreme heat	1.089 (1.020, 1.162)	1.088 (1.019, 1.162)	1.067 (0.996, 1.143)	1.095 (1.021, 1.175)
Cumulative	Extreme cold	1.120 (1.052, 1.193)	1.173 (1.100, 1.250)	1.134 (1.056, 1.217)	1.123 (1.046, 1.205)
	Moderate cold	1.075 (1.026, 1.127)	1.121 (1.068, 1.176)	1.093 (1.036, 1.153)	1.090 (1.033, 1.151)
	Moderate heat	1.073 (1.019, 1.129)	1.069 (1.014, 1.125)	1.052 (0.997, 1.111)	1.072 (1.014, 1.133)
	Extreme heat	1.092 (1.023, 1.165)	1.089 (1.021, 1.163)	1.068 (0.997, 1.144)	1.095 (1.021, 1.174)

[a]Hazard ratios are based on Cox proportional hazards models with average weekly temperatures modeled as time-dependent variables for exposures during four time windows: 1-week (average weekly temperature during the current week), 4-week (temperature during the current and 3 previous weeks), late pregnancy (average weekly temperatures for gestational week 20 through week 36 or birth), and cumulative (average weekly temperatures for gestational week 1 through week 36 or birth). Data for term births are censored after the 36th week of pregnancy. Estimates are relative to the median temperature for the study area (24.4 ^{o}C). The following variables were adjusted for: maternal age, education, and parity, baby's sex, year and month of conception, and relative humidity (during the corresponding time window). [b]Extreme cold, moderate cold, moderate heat and extreme hot were defined as local 1st (7.6 ^{o}C), 5th (11.2 ^{o}C), 95th (30.7 ^{o}C) and 99th (31.9 ^{o}C) percentile temperature over 11-year period, respectively.

Table S2. Cumulative effects of low and high temperatures using quasi-Poisson regression with distributed lag non-linear model.

Temperature[a]	Relative risk (95% confidence interval)[b]		
	Lag 0-6	Lag 0-13	lag 0-27
Extreme cold	1.072 (0.975, 1.178)	1.111 (0.988, 1.249)	1.273 (1.085, 1.494)
Moderate cold	1.028 (0.945, 1.117)	1.069 (0.961, 1.188)	1.246 (1.079, 1.438)
Moderate heat	1.067 (0.992, 1.148)	0.993 (0.907, 1.087)	0.982 (0.871, 1.107)
Extreme heat	1.083 (0.988, 1.186)	0.992 (0.886, 1.110)	0.986 (0.849, 1.456)

[a]Extreme cold, moderate cold, moderate heat and extreme hot were defined as local 1st (7.6 ^{o}C), 5th (11.2 ^{o}C), 95th (30.7 ^{o}C) and 99th (31.9 ^{o}C) percentile temperature over 11-year period, respectively.
[b]Estimates are relative to the median temperature for the study area (24.4 ^{o}C). The following variables were adjusted for: relative humidity (during the corresponding day), long-term trend and seasonality, day of the week, and daily expected count of preterm births on the corresponding day.

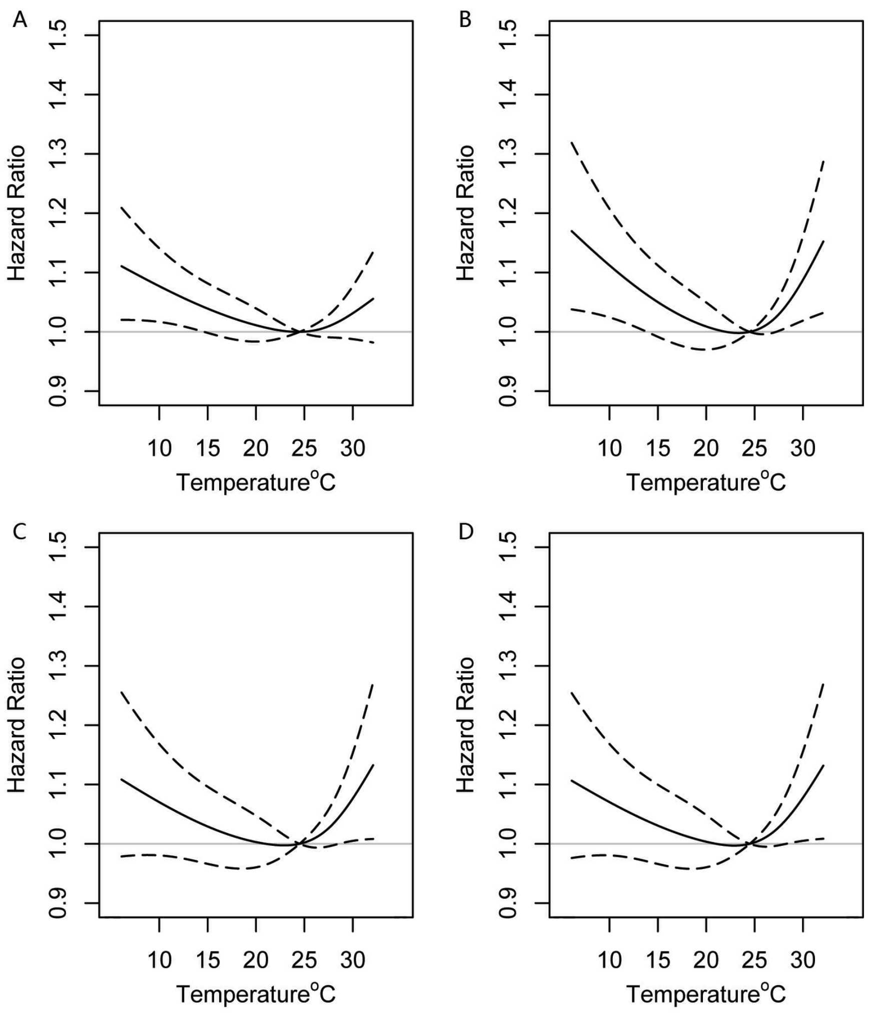

Figure S1. Adjusted hazard ratios (solid line) and 95% confidence intervals (dashed lines) for preterm birth in association with weekly average temperature modeled as a time-dependent variable during 4 time windows after adjustment for PM10, SO2 and NO2 during 2006-2010: 1-week (A), 4-week (B), late pregnancy (C), and cumulative (D). Estimates are relative to the median temperature for the study area (24.4 °C). All values are based on Cox proportional hazards models with gestational age as the underlying time axis and adjusted for maternal age, education, parity, baby's sex, year and month of conception, relative humidity and air pollutants (during the corresponding time window).

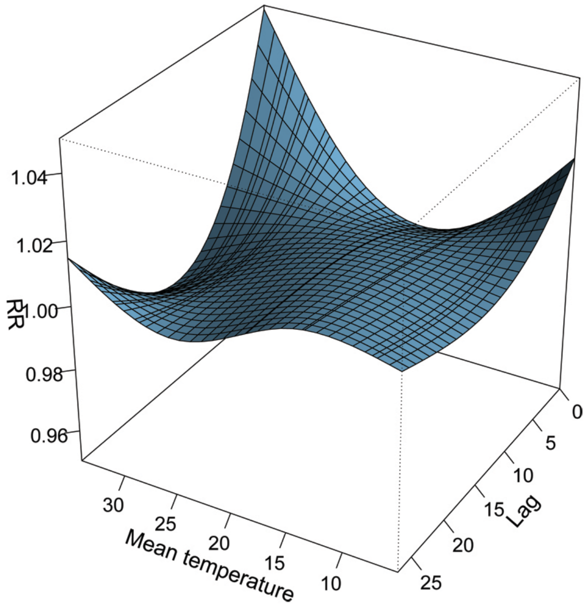

Figure S2. Relationship between mean temperature and preterm birth using quasi-Poisson regression with distributed lag non-linear model. Estimates are relative to the median temperature for the study area (24.4 ^{o}C). The following variables were adjusted for: relative humidity (during the corresponding day), long-term trend and seasonality, day of the week, and daily expected count of preterm births on the corresponding day. Lags represents single-day lags (lag 0, representing temperature on the same day of preterm birth, up to 27-day lag).
